# Multifactorial analysis of willingness to undergo subcutaneous allergen immunotherapy in pediatric patients

**DOI:** 10.12669/pjms.40.5.8581

**Published:** 2024

**Authors:** Bo Ding, Jie Zhong, Chun Jiang, Tian Ma, Qiuzhi Shen, Yanming Lu

**Affiliations:** 1 Bo Ding, Department of Pediatrics, Renji Hospital Affiliated to, Shanghai JiaoTong University School of Medicine, 2000 Jiangyue Road, Shanghai 201112, P.R. China; 2 Jie Zhong, Department of Pediatrics, Renji Hospital Affiliated to, Shanghai JiaoTong University School of Medicine, 2000 Jiangyue Road, Shanghai 201112, P.R. China; 3 Chun Jiang, Department of Pediatrics, Renji Hospital Affiliated to, Shanghai JiaoTong University School of Medicine, 2000 Jiangyue Road, Shanghai 201112, P.R. China; 4 Tian Ma Department of Pediatrics, Ningbo Hangzhou Bay Hospital, Ningbo, Zhejiang Province 315000, China; 5 Qiuzhi Shen, Department of Pediatrics, Renji Hospital Affiliated to, Shanghai JiaoTong University School of Medicine, 2000 Jiangyue Road, Shanghai 201112, P.R. China; 6 Yanming Lu, Department of Pediatrics, Renji Hospital Affiliated to, Shanghai JiaoTong University School of Medicine, 2000 Jiangyue Road, Shanghai 201112, P.R. China

**Keywords:** Multifactorial analysis, Subcutaneous allergen, Immunotherapy, Pediatric

## Abstract

**Objective:**

To explore factors influencing the acceptance of allergen immunotherapy (AIT) for the treatment of allergic respiratory diseases by pediatric patients and their families.

**Methods:**

A total of 406 children (210 males and 196 females) attending the pediatric outpatient clinics and wards of the Renji Hospital, Shanghai Jiao Tong University School of Medicine from June 2020 to April 2022. Those who met the criteria for the AIT treatment, were included in the survey. An online 20-item questionnaire was developed. Data on patient’s general characteristics, allergic disease status, family history of allergies, general family information, parental knowledge of allergic diseases, and whether the AIT treatment was recommended by a physician, were collected. The patients were divided into two groups according to their willingness to receive AIT: a reluctant or neutral group (n = 182), and a willing group (n = 224). A univariate analysis of the willingness to undergo AIT was done to detect parameters that significantly differed between the groups, and the identified factors were used as independent variables in the multifactorial logistic regression analysis.

**Results:**

The severity of allergic disease, presence of drug allergy, occurrence of severe allergic reactions, mother’s education, distance from home to the hospital, parental knowledge of allergic diseases, and whether the doctor recommended AIT were all statistically different between the groups (p < 0.05). Multifactorial logistic regression analysis showed that the degree of allergic rhinitis (AR), or asthma (AS), parental knowledge of allergic diseases, and doctor’s recommendation of AIT were the factors that influenced the willingness of pediatric patients to receive AIT.

**Conclusions:**

The severity of AR and AS, parental knowledge of allergic diseases, and doctor’s recommendation influenced the willingness of pediatric patients to receive AIT.

## INTRODUCTION

Allergic rhinitis (AR) and asthma (AS) are chronic inflammatory diseases caused by airborne allergens (mainly mites) and are mediated by the immunoglobulin IgE.^1^-^3^ In pediatric patients (under 18 years of age), there is a considerable and increasing prevalence of respiratory allergic diseases. A 2019 study showed that there are five million pediatric patients with AR in the USA and approximately 5-10% of children and adolescents are diagnosed with asthma worldwide.^4^ AS and AR seriously affect the quality of life of affected children and is associated with a significant socio-economic burden. Inhaled glucocorticoids (ICS) are the first line of treatment for respiratory allergic diseases and are indicated for almost all children with AS and AR.

However, ICS treatment is associated with systemic adverse effects similar to those seen with oral glucocorticoids, as ICS preparations are continuously updated, treatment cycles are extended (especially in children with other allergic comorbidities), and different hormone preparations are combined (which will result in increased systemic absorption of the drug).^5^ Vidian neurectomy, a common surgical method, is also widely used in the treatment of AR, with an effective rate of ~76.0%. However, it is associated with large trauma, and varying degrees of ocular dry complications in patients.^6^ The World Health Organization (WHO) recommends a “four-in-one” approach to the optimal treatment for respiratory allergic diseases that combines allergen avoidance, pharmacotherapy, allergen immunotherapy (AIT) and patient education.^7^

AIT is an allopathic treatment for IgE-mediated Type-I allergic diseases and is clinically recommended by various guidelines.^8^-^10^ Adding AIT into the treatment regimen can help the patients to build immune tolerance, reduce both the symptoms and the need for symptomatic drug therapy. However, it is still not routinely used worldwide,^11^,^12^ and the acceptance rate of AIT among pediatric patients is low.^13^ Factors, such as the family situation of the patient, parental knowledge of allergic diseases, physician’s understanding of AIT may all influence the willingness of pediatric patients to receive AIT. We aimed to provide a clinical basis and reference for promoting the widespread use of AIT in pediatric clinics.

## METHODS

Five hundred children attending the pediatric outpatient clinics and wards of the Renji Hospital, Shanghai Jiao Tong University School of Medicine from June 2020 to April 2022, who met the criteria for the AIT treatment, were included in the survey. An electronic questionnaire (patient’s version of the AIT intention survey)^14^ was distributed to the guardians of the pediatric patients.

### Ethical Approval:

The ethical committee of Shanghai Jiaotong University School of Medicine, Renji Hospital Ethics Committee approved this study on June 30^th^ 2023, No. KY2023-042-B.

### Inclusion & Exclusion criteria:

Patients aged five years and above, patients with respiratory allergic diseases, presence of informed family consent were included while patients with severe cardiovascular and immunological diseases, and children with severe psychological disorders were excluded.

### Research Methodology:

The online questionnaire was developed based on the literature and guidelines on respiratory allergic diseases and AIT in children,^15^ and reviewed and revised in consultation with relevant pediatric experts. The questionnaire consisted of 20 entries on the following: patient’s gender, age, allergic disease status (diagnosis, type of allergen, degree of rhinitis effect, degree of asthma effect, presence of adenoid hypertrophy, presence of food allergy, presence of drug allergy, whether severe allergic reaction occurred), family history of allergies (whether the parents are allergic), general family situation (parental age and education level, annual family income, distance from home to the hospital), parental knowledge of allergic diseases, whether the AIT treatment was recommended by a physician.

The online questionnaire was administered via mobile phone to patients who met the inclusion criteria. The purpose of the survey was explained to the respondents by uniformly trained physicians prior to the survey. The meaning of each item in the questionnaire was fully explained; each item was a compulsory single choice question. The completed questionnaires were collected at the end of the survey.

### Mediation analysis:

Mediation analysis was conducted to investigate the pathways through which various factors influence the willingness of paediatric patients to receive allergen immunotherapy (AIT). The analysis focused on assessing how the recommendation by doctors mediates the relationship between several key factors (such as the impact of rhinitis, asthma, parental awareness, drug allergy, and severe allergic reactions) and the patients’ willingness to undergo AIT.

We employed a causal mediation analysis framework to quantify the direct and indirect effects of the aforementioned factors on the willingness to receive AIT. The analysis was performed using the “*paramed*” package in STATA, which is designed for causal mediation analysis and allows for the estimation of direct, indirect, and total effects in the presence of a mediator variable.

For each factor considered, logistic regression models were fitted to estimate the controlled direct effect (CDE), natural direct effect (NDE), natural indirect effect (NIE), and marginal total effect (MTE). The outcome variable (Y) was the willingness to receive AIT. The mediator variable (M) was the doctors’ recommendation for AIT. Each factor was treated as an independent variable (X) in separate models.

The CDE represents the effect of the independent variable on the outcome, controlling for the mediator. The NDE reflects the effect of the independent variable on the outcome that is not mediated by the mediator. The NIE captures the effect of the independent variable on the outcome that operates through the mediator. The MTE is the total effect, encompassing both direct and indirect pathways. The direct acyclic graph (DAG) was generated through Dagitty software version 3.1.

### Statistical analysis:

Excel software was used to create the database, and SPSS 25.0 software was used for data processing and analysis. Count data were expressed as frequencies and percentages (%). Comparisons between groups were made using the chi-square test. The analysis of factors influencing the receipt of AIT treatment was performed using multi-factor logistic regression analysis, and differences were considered statistically significant at P < 0.05.

## RESULTS

**Fig.1:**
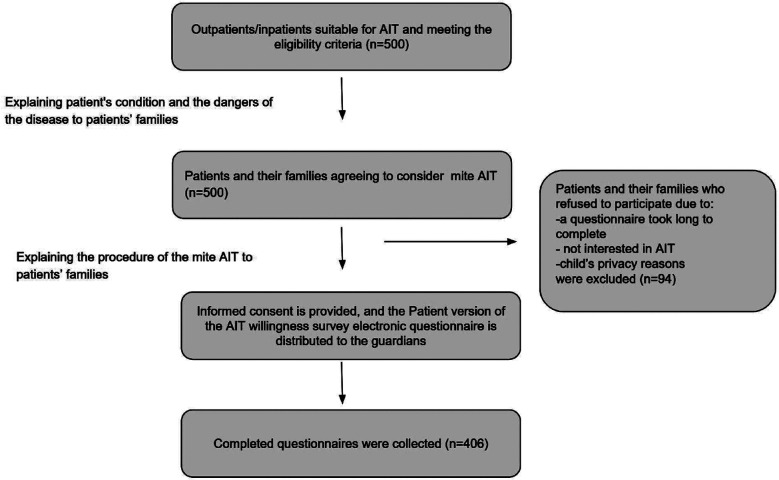
Flowchart of the study.

Of 500 pediatric patients who met the eligibility criteria, 406 (210 males and 196 females) completed questionnaires and were included in the study (Fig.1). The remaining 94 patients withdrew from the study for various reasons, such as: a questionnaire took long to complete; family not interested in AIT; child’s privacy reasons, etc.

The patients were divided into two groups according to the willingness to undergo AIT: a reluctant or neutral group (n = 182), and a willing group (n = 224). As summarized in Table-I, the degree of impact of rhinitis, asthma, whether drug allergy was present, whether severe allergic reactions occurred, mother’s education, distance from home to the hospital, parental knowledge of allergic diseases, and whether the doctor recommended the AIT were all statistically different between the groups (p < 0.05).

**Table-I:** Univariate analysis of pediatric patients’ willingness to undergo AIT.

	Research factors	Reluctance or neutrality	Willing	χ2	P
Gender	Male	88 (48.4%)	122 (54.5%)	1.503	0.22
	Female	94 (51.6%)	102 (45.5%)		
Age (years)	<12 years	95 (52.2%)	105 (46.9%)	1.138	0.286
	12—18 years	87 (47.8%)	119 (53.1%)		
Allergens	Single allergens	48 (26.4%)	55 (24.6%)	0.176	0.675
	Multiple allergens	134 (73.6%)	169 (75.4%)		
Diagnosis	Rhinitis	83 (45.6)	101 (45.1)	5.485	0.241
	asthma	19 (10.4)	33 (14.7)		
	Rhinitis + Asthma	70 (38.5)	86 (38.4)		
	Dermatitis	7 (3.8)	3 (1.3)		
	others	3 (1.6)	1 (0.4)		
The degree of impact of rhinitis	None	67 (36.8)	15 (6.7)	196.59	0.000
	Slight	102 (56.0)	38 (17.0)		
	Moderate	9 (4.9)	150 (67.0)		
	Severe	4 (2.2)	21 (9.4)		
The degree of impact of asthma	None	114 (62.6)	41 (18.3)	120.91	0.000
	Slight	49 (26.9)	46 (20.5)		
	Moderate	16 (8.8)	123 (54.9)		
	Severe	3 (1.6)	14 (6.3)		
Adenoid hypertrophy	No	46 (25.3)	74 (33)	2.93	0.231
	Yes, without surgery	131 (72)	145 (64.7)		
	Yes, with surgery	5 (2.7)	5 (2.2)		
Food allergies	No	81 (44.5)	110 (49.1)	0.853	0.356
	Yes	101 (55.5)	114 (50.9)		
Drug allergy	No	40 (22)	75 (33.5)	6.546	0.011
	Yes	142 (78)	149 (66.5)		
Severe allergic reactions	No	51 (28)	86 (38.4)	4.831	0.028
	Yes	131 (72)	138 (61.6)		
Father’s age	20-30	4 (2.2)	7 (3.1)	2.491	0.477
	30-40	130 (71.4)	145 (64.7)		
	40-50	47 (25.8)	69 (30.8)		
	50-60	1 (0.5)	3 (1.3)		
Father’s education level	Primary school and below	0 (0)	0 (0)	5.696	0.223
	Secondary school	8 (4.4)	6 (2.7)		
	High school	25 (13.7)	33 (14.7)		
	Specialist	47 (25.8)	50 (22.3)		
	Undergraduate	82 (45.1)	93 (41.5)		
	Postgraduate and above	20 (11)	42 (18.8)		
Mother’s age	20-30	4 (2.2)	10 (4.5)	3.22	0.359
	30-40	147 (80.8)	172 (76.8)		
	40-50	30 (16.5)	42 (18.8)		
	50-60	1 (0.5)	0 (0)		
Mother’s education level	Primary school and below	22 (12.1)	1 (0.4)	38.106	0.000
	Secondary school	27 (14.8)	22 (9.8)		
	High school	34 (18.7)	48 (21.4)		
	Specialist	29 (15.9)	67 (29.9)		
	Undergraduate	61 (33.5)	65 (29)		
	Postgraduate and above	9 (4.9)	21 (9.4%)		
Annual household income	<100,000	11 (6)	17 (7.6)	0.814	0.846
	100,000 - 200,000	53 (29.1)	70 (31.3)		
	200,00 - 400,000	54 (29.7)	60 (26.8)		
	>400,000	64 (35.2)	77 (34.4)		
Distance from home to treatment hospital	<0.5 hour	123 (67.6%)	146 (65.2%)	12.58	0.002
	0.5-1 hour	31 (17%)	63 (28.1%)		
	>1 hour	28 (15.4%)	15 (6.7%)		
Whether the father is allergic	No	71 (39)	85 (37.9)	0.048	0.826
	Yes	111 (61)	139 (62.1)		
Whether the mother is allergic	No	83 (45.6)	108 (48.2)	0.275	0.6
	Yes	99 (54.4)	116 (51.8)		
Parental awareness of the disease	No treatment required	77 (42.3)	14 (6.3)	91.308	0
	Need for medication	93 (51.1)	139 (62.1)		
	Requires medication + AIT	12 (6.6)	71 (31.7)		
Do doctors recommend	No	110 (60.4)	17 (7.6)	130.747	0
	Yes	72 (39.6)	207 (92.4)		

(Criteria for evaluating the degree of impact of rhinitis and asthma on life: none: no impact, slight: symptoms are mild and easily tolerated; moderate: symptoms are noticeable and annoying but tolerable; severe: symptoms are intolerable and interfere with daily life and sleep).

### Factors influencing willingness to undergo AIT:

We next used factors that were statistically significant between the groups as independent variables in the multifactorial logistic regression analysis. The results showed that the degree of rhinitis, the degree of asthma, parental knowledge of allergic diseases, and doctor’s recommendation of AIT were the factors that influenced the willingness of pediatric patients to receive AIT (Table-II).

**Table-II:** Multi-factorial Logistic Regression Analysis of Child Patients Receiving AIT.

Research factors	B	S.E.	P value	Exp(B)	95%CI
Any drug allergies	-0.108	0.662	0.870	0.897	0.245-3.283
Any serious allergic reactions	0.420	0.625	0.501	0.657	0.193-2.235
***The degree of impact of rhinitis***			0.000		
None	-1.954	0.755	0.010	0.142	0.032-0.623
Slight	-1.333	0.690	0.053	0.264	0.068-1.020
Moderate	1.716	0.741	0.021	5.562	1.301-23.779
***The degree of impact of asthma***			0.000		
None	-2.513	1.146	0.028	0.081	0.009-0.766
Slight	-1.217	1.137	0.284	0.296	0.032-2.746
Moderate	0.315	1.135	0.781	1.370	0.148-12.676
***Mother’s education level***			0.253		
Primary school and below	-1.484	2.058	0.471	0.227	0.004-12.813
Secondary school	1.537	0.878	0.080	4.650	0.833-25.972
High school	1.454	0.770	0.059	4.281	0.946-19.366
Specialist	1.376	0.711	0.053	3.959	0.982-15.959
Undergraduate	0.956	0.685	0.163	2.602	0.679-9.974
***Distance from home to treatment hospital***			0.732		
<0.5 hour	0.492	0.635	0.439	1.635	0.471-5.674
0.5-1 hour	0.488	0.706	0.490	1.628	0.408-6.492
***Parental awareness of the disease***			0.000		
No treatment required	-2.754	0.629	0.000	0.064	0.019-0.219
Need for medication	-1.959	0.555	0.000	0.141	0.048-0.418
Does the doctor recommend desensitisation treatment	1.658	0.505	0.001	5.247	1.952-14.107

### Mediation Analysis:

**Fig.2:**
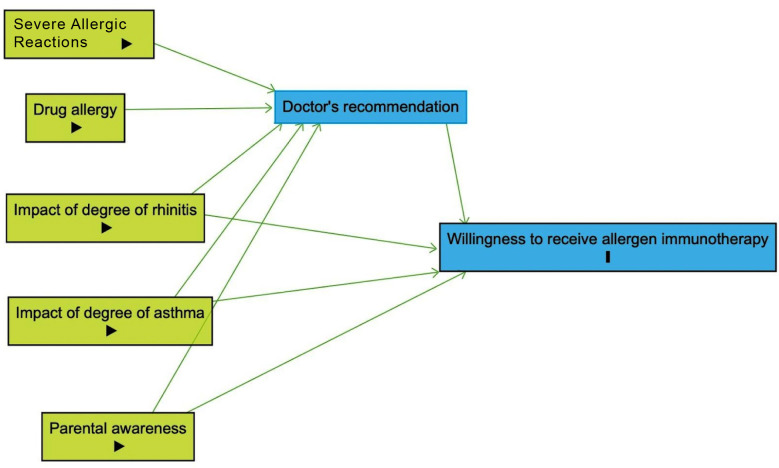
Direct acyclic graph depicting the causal pathway determining the willing to receive allergen immunotherapy via potential exposure and mediator. Green coloured boxes indicate the exposure variables, where the severe drug allergy and drug allergy are completed mediated through doctor’s recommendation without any direct effect, while the impact of degree of rhinitis, asthma, parental awareness has partial mediation via doctor’s recommendation and also has natural direct effect on willingness to receive allergen immunotherapy.

Our mediation analysis revealed the influence of several factors on the willingness to receive AIT, mediated through doctors’ recommendations (Table-III, Fig.2).

**Table-III:** Mediation Analysis of Factors Influencing Willingness to Receive Allergen Immunotherapy via Doctors’ Recommendation.

Mediation Pathway	Controlled Direct Effect (CDE) with 95%CI	Natural Direct Effect (NDE) with 95%CI	Natural Indirect Effect (NIE) with 95%CI	Marginal Total Effect (MTE) with 95%CI
Impact of Rhinitis on Willingness (via Doctors’ Recommendation)	26.25 (13.34-51.66)	26.25 (13.34-51.66)	1.72 (1.51-1.97)	45.29 (22.68-90.44)
Impact of Asthma on Willingness (via Doctors’ Recommendation)	10.71 (5.78-21.23)	10.71 (5.78-21.23)	1.50 (1.36-1.71)	16.12 (8.81-31.88)
Parental Awareness on Willingness (via Doctors’ Recommendation)	8.25 (4. 16-16.35)	8.25 (4.16-16.35)	1.76 (1.41-2.20)	14.52 (7.05-29.90)
Drug Allergy on Willingness (via Doctors’ Recommendation)	1.30 (0.77-2.21)	1.30 (0.77-2.21)	0.68 (0.61-0.76)	0.89 (0.52-1.52)
Severe Allergic Reactions on Willingness (via Doctors’ Recommendation)	1.37 (0.82-2.29)	1.37 (0.82-2.29)	0.70 (0.63-0.78)	0.96 (0.57-1.62)

### Impact of Rhinitis:

The controlled direct effect (CDE) and natural direct effect (NDE) of the impact of rhinitis on willingness to receive AIT, both mediated by doctors’ recommendations, were significant with coefficients of 26.25 (95% CI: 13.34-51.66). The natural indirect effect (NIE) was 1.72 (95% CI: 1.51-1.97), and the marginal total effect (MTE) was 45.29 (95% CI: 22.68-90.44). This suggests a partial mediation, indicating that doctors’ recommendations partially mediate the relationship between the impact of rhinitis and the willingness to receive AIT.

### Impact of Asthma:

The CDE and NDE for asthma were 10.71 (95% CI: 5.78-21.23), with an NIE of 1.50 (95% CI: 1.36-1.71) and an MTE of 16.12 (95% CI: 8.81-31.88). This also points to partial mediation, where the decision to undergo AIT is influenced by both the direct impact of asthma and the mediating effect of doctors’ recommendations.

### Parental Awareness:

The mediation effect of parental awareness showed a CDE and NDE of 8.25 (95% CI: 4.16-16.35), an NIE of 1.76 (95% CI: 1.41-2.20), and an MTE of 14.52 (95% CI: 7.05-29.90). This indicates that while parental awareness directly influences the willingness for AIT, doctors’ recommendations also play a significant mediating role.

### Drug Allergy:

For drug allergy, the results indicated a full mediation by doctors’ recommendations. The CDE and NDE were 1.30 (95% CI: 0.77-2.21), and the NIE was 0.68 (95% CI: 0.61-0.76), with an MTE of 0.89 (95% CI: 0.52-1.52). The full mediation suggests that the influence of drug allergy on the willingness to undergo AIT is predominantly through the pathway of doctors’ recommendations.

### Severe Allergic Reactions:

Similar to drug allergy, severe allergic reactions showed a full mediation effect with a CDE and NDE of 1.37 (95% CI: 0.82-2.29), an NIE of 0.70 (95% CI: 0.63-0.78), and an MTE of 0.96 (95% CI: 0.57-1.62). This indicates that the decision-making process is significantly influenced by the recommendations of doctors, rather than directly by the severity of allergic reactions.

## DISCUSSION

In this paper, we analyzed the factors influencing the willingness of 406 pediatric patients to undergo AIT and identified the degree of impact of rhinitis or asthma, parental awareness of allergic diseases, and whether the doctor recommends the AIT treatment as influencing factors for the acceptance of AIT in pediatric patients. Our results showed that the degree of impact of rhinitis and of asthma, parental awareness of allergic diseases and whether the doctor recommended the desensitization treatment were all factors that influenced the willingness of pediatric patients to undergo AIT.

The clinical effectiveness of AIT depends on the adequate doses and sufficient treatment duration.^16^ Therefore, poor compliance may present a significant problem, similar to other long-term treatments.^17^ Our results showed that the degree of impact of AR and asthma influenced the willingness of pediatric patients to undergo AIT. These observations are in agreement with the previous study by Damm et al., that showed that allergy patients suffering from AR symptoms have the strongest preference for AIT.^18^ Similarly, Ciprandi et al.^19^ reported that higher rates of AIT treatment were associated with longer AR duration (7.2 vs. six years), and asthma was present in 41% of AIT-treated patients and in 34% of remaining patients.

In our study, the proportion of children with AR, willing to receive AIT, increased from 23.9% to 92.9% as the level of impact of rhinitis increased from “slightly or less” to “moderately” and above. Patients with mild AR have an insignificant decline in quality of life, while those with moderate to severe disease have a severe decline in the quality of life.^20^ Studies have shown that even after the treatment, around one in 10 patients still have significant symptoms and around 40% of patients with AR have disrupted sleep, work and school.^21^ Similarly, the proportion of children with AS, willing to receive AIT, increased from 34.8% to 87.8% as the level of impact of asthma increased from “slightly or less” to “moderately” or more. According to the Pediatric Asthma Quality of Life Questionnaire (PAQLQ), the quality-of-life score of children with asthma decreases significantly only when the number of annual wheezing episodes is ≥4. 22

This may explain to some extent our observation of higher AIT acceptance rate in children with AR compared to patients with AS. In addition, respiratory allergic diseases are closely associated with acute respiratory infectious diseases^23^ and concomitant conditions such as food allergy and hyperactivity disorders.^24^,^25^ Therefore, respiratory allergic diseases are often associated with recurrent episodes of multiple diseases, prolonged treatment cycles and increased risk of adverse drug reactions that impact children’s growth and development. We may speculate that patients suffering from the disease, especially during an acute attack, may be more willing to undergo a combination of treatments, including AIT in order to achieve prolonged remission and improve the quality of life.

Our study demonstrated that only 83 out of 406 families of children with respiratory allergic diseases in our study believed that a combination of medication and AIT therapy was needed, a correct awareness rate of only 20.4%. As parents’ perception of allergic diseases changed from “no treatment needed” to “comprehensive treatment including medication + AIT needed”, the proportion of patients willing to receive AIT increased from 47.7% to 85.5%. A previous study by Calderon et al.^26^ that assessed patients’ understanding of allergy and AIT acceptance showed that almost third of the participants did not know for which allergy they were being treated, and around 40% of the patients reported a perception of low effectiveness. The study reported introducing a new communication template on allergy and allergen immunotherapy and showed that it allowed patients participating in the survey to feel better informed and more likely to initiate or complete AIT.^26^ Together with our observations, these results suggest that the lack of knowledge about the management of respiratory allergic diseases and attitudes and understanding of AIT play an important role in adherence to AIT treatment. It is important to educate families about the prevention and treatment of respiratory allergic diseases and to promote the “four-in-one” approach.

The proportion of patients willing to undergo AIT increased from 13.4% to 74.2% when the AIT treatment was recommended by a physician. Previous study by Nam et al. that focused on attitudes and satisfaction of 167 patients who underwent AIT, showed that more than half (68.7%) initiated AIT based on their doctor’s recommendation.^27^ Physicians need to use certain experience, skills and knowledge to recommend AIT treatment to patients in a way that will be understood by pediatric patients and their family members. AIT compliance can be further improved when both parties will have more communication to discuss the child’s condition, benefits/risks of treatment, impact of treatment on daily life, etc.^28^ Although 55.1% of the patients in this study said they would accept AIT, only 25% of them actually did. The reasons for this discrepancy were related to the physician’s own knowledge of AIT, the high cost of AIT drugs and the fact that they were not covered by the health insurance, and the long treatment period.

The results of the mediation analysis in our study highlight the critical role of doctors’ recommendations in mediating the decision to opt for AIT among pediatric patients, particularly in the context of drug allergies and severe allergic reactions where it acts as a full mediator. For rhinitis, asthma, and parental awareness, the mediation was partial, suggesting that these factors independently contribute to the decision alongside the influence exerted through doctors’ recommendations.

### Limitations:

Our study has some limitations, mainly related to the observational, self-reported, nature of the internet-based surveys. Self-assessments of symptom severity and impact on quality of life were not recorded using standardized, validated tools, which may introduce variability and impact the accuracy of our results. Additionally, the baseline level of awareness of AIT may vary among the physicians to another and depend on the specialty. Furthermore, the study questionnaire was not extensively tested or psychometrically validated.

## CONCLUSION

Our study showed that the severity of AR and AS, parental knowledge of allergic diseases, and doctor’s recommendation of AIT influenced the willingness of pediatric patients to receive AIT. Currently, most patients have a misconception about the treatment of respiratory allergic diseases, believing that they do not require treatment or that medication alone is sufficient. There is a need to increase the awareness of allergic diseases among pediatric parents and their families. The physicians need to find understandable ways to convey their message and recommend AIT treatment to the right patients. Improved compliance with AIT treatment will allow clinicians and patients to achieve good control of respiratory allergic diseases. Our results further strengthen the importance of ensuring that patients and their families receive full information about the benefits of AIT in prevention and treatment of respiratory allergic diseases.

### Authors’ contributions:

**BD:** Conceived and designed the study.

**JZ**, **CJ**, **TM**, **QS** and **YL:** Collected the data and performed the analysis.

**BD:** Was involved in the writing of the manuscript and is responsible for the integrity of the study.

All authors have read and approved the final manuscript.

## References

[1] Shamji MH, Sharif H, Layhadi JA, Zhu R, Kishore U, Renz H (2022). Diverse immune mechanisms of allergen immunotherapy for allergic rhinitis with and without asthma. J Allergy Clin Immunol.

[2] Passalacqua G, Landi M, Peroni DG (2020). Allergen immunotherapy for pediatric asthma:current evidence and knowledge gaps. Curr Opin Allergy Clin Immunol.

[3] Ciprandi G, Puccinelli P, Incorvaia C, Passalacqua G, Italian Cometa Study Group (2017). The relevance of house dust mites allergy in clinical practice:the epidemiological impact on allergen immunotherapy. Immunotherapy.

[4] Centers for Disease Control and Prevention Allergies and hay fever fast stats.

[5] Respiratory Group of Pediatric Branch of Shanghai Medical Association (2021). Expert consensus on identification and prevention of adverse reactions of commonly used asthma drugs in children. Chin J Appl Clin Pediatr.

[6] Wu R, Dong L, Mao H, Wang J, Ma D, Sun J (2022). Clinical study on the treatment of moderate to severe persistent allergic rhinitis by posterior nasal nerve combined with anterior ethmoid neurotomy. Pak J Med Sci.

[7] Yang L, Zhu R (2017). Immunotherapy of house dust mite allergy. Hum Vaccin Immunother.

[8] Otolaryngology Professional Committee, Pediatrician Branch, Chinese Medical Doctor Association (2019). Clinical Practice Guideline:Diagnosis and Treatment in Children with Allergic Rhinitis. Chin J Pract Pediatr.

[9] Pajno GB, Bernardini R, Peroni D, Arasi S, Martelli A, Landi M (2017). Clinical practice recommendations for allergen-specific immunotherapy in children:the Italian consensus report. Ital J Pediatr.

[10] Xiang L, Zhao J, Bao Y, Shao J, Liu C, Li M (2018). Expert consensus on mite specific immunotherapy for airway allergic diseases in children. Chin J Appl Clin Pediatr.

[11] Shin YS, Jung JW, Park JW, Choi JH, Kwon JW, Lee S (2019). Clinical Efficacy of Allergen-Specific Immunotherapy from Patient and Physician Perspectives. Yonsei Med J.

[12] Rael E (2016). Allergen Immunotherapy. Prim Care.

[13] Anolik R, Schwartz AM, Sajjan S, Allen-Ramey F (2014). Patient initiation and persistence with allergen immunotherapy. Ann Allergy Asthma Immunol.

[14] Electronic Questionnaire (patient's version of the AIT intention survey).

[15] Alvaro-Lozano M, Akdis CA, Akdis M, Alviani C, Angier E, Arasi S (2020). Allergen Immunotherapy in Children User's Guide. Pediate Alleryg Immu.

[16] Cox L, Nelson H, Lockey R, Calabria C, Chacko T, Finegold I (2011). Allergen immunotherapy:a practice parameter third update. J Allergy Clin Immunol.

[17] Burkhart PV, Sabaté E (2003). Adherence to long-term therapies:evidence for action. J Nurs Scholarsh.

[18] Damm K, Volk J, Horn A, Allam JP, Troensegaard-Petersen N, Serup-Hansen N (2016). Patient preferences in allergy immunotherapy (AIT) in Germany - a discrete-choice-experiment. Health Econ Rev.

[19] Ciprandi G, Natoli V, Puccinelli P, Incorvaia C, Italian Cometa Study Group (2017). Allergic rhinitis:the eligible candidate to mite immunotherapy in the real world. Allergy Asthma Clin Immunol.

[20] Fu M, Luo J (2021). Effect of specific immunotherapy on the quality of life of patients with allergic rhinitis. Int J Otolaryngol Head Neck Surg.

[21] Chen H, Hu H, Song C, Liu X, Yin W, Li X (2019). Analysis of the uncontrolled rate of allergic rhinitis and its associated factors. Chin Arch Otolaryngol Head Neck Surg.

[22] Liu Y, Chen N, Hong A, Yuan C (2022). Analysis of the current status and factors influencing self-reported quality of life in outpatient children and adolescents with bronchial asthma. J Nurs Trai.

[23] Dubakiene R, Rubinaitė V, Petronytė M, Dalgediene I, Rudzeviciene O, Dubakaite D (2017). Investigation of markers of allergic sensitization and viral infections in children with allergy and asthma. Acta Med Litu.

[24] Zhang X, Guo Z (2020). Exploring the correlation between food allergy and asthma in children. J Clin Pulm Med.

[25] Shyu CS, Lin HK, Lin CH, Fu LS (2012). Prevalence of attention-deficit/hyperactivity disorder in patients with pediatric allergic disorders:a nationwide, population-based study. J Microbiol Immunol Infect.

[26] Calderon MA, Cox L, Casale TB, Mosges R, Pfaar O, Malling HJ (2015). The effect of a new communication template on anticipated willingness to initiate or resume allergen immunotherapy:an internet-based patient survey. Allergy Asthma Clin Immunol.

[27] Nam YH, Lee SK (2017). Physician's recommendation and explanation is important in the initiation and maintenance of allergen immunotherapy. Patient Prefer Adherence.

[28] European Patients Forum Adherence and Concordance.

